# Comparison of routine field epidemiology and whole genome sequencing to identify tuberculosis transmission in a remote setting

**DOI:** 10.1017/S0950268820000072

**Published:** 2020-02-04

**Authors:** J. L. Guthrie, L. Strudwick, B. Roberts, M. Allen, J. McFadzen, D. Roth, D. Jorgensen, M. Rodrigues, P. Tang, B. Hanley, J. Johnston, V. J. Cook, J.L. Gardy

**Affiliations:** 1 School of Population and Public Health, University of British Columbia, Vancouver, Canada; 2 Yukon Communicable Disease Control, Health and Social Services, Government of Yukon, Whitehorse, Canada; 3 British Columbia Centre for Disease Control, Vancouver, Canada; 4 British Columbia Centre for Disease Control, Public Health Laboratory, Vancouver, Canada; 5 Department of Pathology, Sidra Medical and Research Center, Doha, Qatar; 6 Department of Health and Social Services, Government of Yukon, Whitehorse, Canada; 7 Department of Medicine, University of British Columbia, Vancouver, Canada

**Keywords:** Epidemiology, programme assessment, transmission, tuberculosis (TB), whole genome sequencing

## Abstract

Yukon Territory (YT) is a remote region in northern Canada with ongoing spread of tuberculosis (TB). To explore the utility of whole genome sequencing (WGS) for TB surveillance and monitoring in a setting with detailed contact tracing and interview data, we used a mixed-methods approach. Our analysis included all culture-confirmed cases in YT (2005–2014) and incorporated data from 24-locus Mycobacterial Interspersed Repetitive Units-Variable Number of Tandem Repeats (MIRU-VNTR) genotyping, WGS and contact tracing. We compared field-based (contact investigation (CI) data + MIRU-VNTR) and genomic-based (WGS + MIRU-VNTR + basic case data) investigations to identify the most likely source of each person's TB and assessed the knowledge, attitudes and practices of programme personnel around genotyping and genomics using online, multiple-choice surveys (*n* = 4) and an in-person group interview (*n* = 5). Field- and genomics-based approaches agreed for 26 of 32 (81%) cases on likely location of TB acquisition. There was less agreement in the identification of specific source cases (13/22 or 59% of cases). Single-locus MIRU-VNTR variants and limited genetic diversity complicated the analysis. Qualitative data indicated that participants viewed genomic epidemiology as a useful tool to streamline investigations, particularly in differentiating latent TB reactivation from the recent transmission. Based on this, genomic data could be used to enhance CIs, focus resources, target interventions and aid in TB programme evaluation.

## Introduction

Tuberculosis (TB) remains an important public health concern in Canada, particularly in Northern rural and remote areas where the endemic spread of TB is commonplace [1, 2]. Understanding the patterns of transmission in these settings is an integral part of developing evidence-based prevention and care strategies, and prioritizing public health resources. This includes understanding the burden of disease resulting from recent local transmission *vs.* reactivation of historic latent TB infection (LTBI), as well as understanding the nature of recent transmission. This latter point is critical for improving TB services in a region – understanding the clinical, demographic and/or epidemiological factors driving TB transmission is vital to developing informed prevention programmes, screening activities and contact investigations (CIs), and ultimately preventing the continued spread of TB.

Field-based epidemiologic investigation is used to identify both infected contacts, secondary active cases and possible sources of a given case, and for decades was the only means to detect transmission [3]. In recent years, a combination of field and molecular epidemiology has been used in many settings – contact data collected through patient interviews may reveal the potential links between cases, while genotyping techniques identify related *Mycobacterium tuberculosis* (*Mtb*) isolates and can help to confirm or refute a potential transmission event. Now, several studies have shown that whole genome sequencing (WGS) yields more accurate transmission reconstructions than the approaches based on genotypic data [4–8].

Despite global interest in WGS as a tool for understanding TB epidemiology and a continuously expanding dataset of publicly available *Mtb* genomes, there are gaps in our understanding of how useful this new technique is. There are technical questions around how consistent *Mtb* mutation rates are, particularly during latent infection *vs.* active disease [9, 10] and from human host to human host [11, 12], as well as around how to identify transmission-informative variants in the many repetitive elements within the *Mtb* genome [13 14]. There are also questions surrounding its utility. In rural and remote settings of low-incidence countries, where detailed contact tracing and interview data are often available for each case, it is not known whether WGS offers any benefit over the current standard of care – interpreting genotyping data in the context of this rich field epidemiological data – and there have only been limited comparisons of how useful the molecular data alone is, whether genotypic or genomic [1, 15]. Furthermore, there has been no qualitative feedback data from frontline public health personnel describing if/how molecular data improved their ability to understand a cluster of cases in a remote setting.

The Yukon Territory (YT), located in Canada's Northwest, has a higher TB incidence (12.1 per 100 000) than the Canadian average (4.9 per 100 000), but lower than other Northern Canadian settings [16, 17]. The majority of YT residents diagnosed with TB are Canadian-born (93.8%) and live in remote regions (84.4%) [18]. All YT TB cases are managed by a small team of public health professionals, many of whom have deep and long-standing ties to the territory; this strong tradition of engagement between TB nurses, community nurses and YT's communities means the local communicable disease unit has uniquely detailed insights into the social networks underlying YT's TB clusters. These close ties, coupled to a small, remote population with little in- or out-migration, make YT an ideal region in which the utility of genomic data in enhancing CI is explored. Here, we compare WGS to 24-locus Mycobacterial Interspersed Repetitive Units-Variable Number of Tandem Repeats (MIRU-VNTR) coupled to robust traditional field epidemiology to inform TB transmission, outbreaks and reactivation of LTBI. We recently reported [18] the results of these reconstructions. In this study, we present the comparison of our two different approaches as well as qualitative user feedback on the utility of genotyping and genomics in a remote setting with a comprehensive TB CI programme.

## Methods

### Study setting and design

Our study took place in YT, Canada, a remote arctic/sub-arctic territory with a study population that included all 32 persons diagnosed in YT with culture-confirmed TB from 2005 through 2014 (84.2% of all 38 diagnoses). Yukon Communicable Disease Control (YCDC), in partnership with Community Nursing, is responsible for patient care and treatment, with contracted TB services including laboratory diagnostics, case management support and access to a shared data system provided by the BC Public Health Laboratory (BCPHL) and the British Columbia Centre for Disease Control (BCCDC).

### Bacterial culture, genotyping and whole genome sequencing

All *Mtb* isolates were obtained from specimens submitted to BCPHL for routine clinical testing of TB. *Mtb* isolates were cultured, DNA extracted and 24-locus MIRU-VNTR genotyping was carried out using standard methods [2, 19]. All samples were sequenced on the Illumina HiSeq2500 platform (Illumina, San Diego, USA) at the Michael Smith British Columbia Genome Sciences Centre (Vancouver, Canada) to produce 125-bp paired-end reads, which were mapped to the H37Rv reference genome (GenBank ID: NC000962.2) using the Public Health England/Oxford University bioinformatics pipeline [20].

### Source case identification

Two independent teams – one working with MIRU-VNTR and detailed CI data and the other working with WGS data and basic clinical and epidemiological information – each reconstructed the most likely transmission pathways for each culture-positive TB case diagnosed in YT from 2005 to 2014. The teams then met to jointly infer the most plausible transmission networks, given both the social CI and WGS data.

#### Source identification by field and molecular epidemiology

The first team (field-based) comprised YCDC nursing staff and programme managers responsible for the treatment and care of all TB patients and their contacts in the territory. They reviewed detailed notes from CIs for each individual in the study, and were provided with the MIRU-VNTR cluster for each isolate, along with a general description of each cluster across BC and YT (e.g. size, geographic distribution, basic demographics) (Supplementary Fig. S1). The team was provided with a structured spreadsheet and were asked to identify each case's most likely source from the following options: a specific individual within YT, an unknown individual within YT, acquisition from an unknown individual through travel outside YT or reactivation of LTBI acquired prior to the study period. Deliberations took into consideration MIRU-VNTR data, along with each TB case's prior contact history, past travel/residence, symptom onset date, tuberculin skin test records and transmission risk factors including acid-fast bacillus smear status and presence of cavitary disease. Respondents were also asked to provide a confidence score to each presumed source: 0 – not at all confident, 1 – somewhat confident, 2 – very confident, 3 – certain.

#### Source identification by genomic epidemiology

The second team (genomic-based), comprising TB genomics experts from BCCDC, had access to MIRU-VNTR and WGS data for each YT isolate, as previously described [18], as well as WGS data from all MIRU-VNTR clustered *Mtb* isolates from cases diagnosed in BC as part of a 10-year retrospective study [2], including those that matched at least 23/24 MIRU-VNTR loci with a YT isolate. Genomic clusters were defined using a threshold of five single nucleotide variants (SNVs) [6] and were assigned a unique identifier (WClustID). Using WGS data but no field epidemiological information, this team independently constructed putative transmission networks from the genome sequences of all YT study isolates (*n* = 32) and any BC isolates within five SNVs of a YT isolate (*n* = 101). A minimum-spanning tree was generated and coloured by MIRU-VNTR cluster ID (MClustID), with labels indicating the genomic WClustID. The team subsequently refined each network with basic case-level data routinely entered into the TB registry as standardised fields, including diagnosis date, area of residence, acid-fact bacilli smear status, chest radiology results and risk factors (HIV, substance use, alcohol misuse). The genomic-based team did not have access to any CI or social network information. The team identified each case's most likely source from the same options and confidence scale as the field-based team as described above.

#### Source identification consensus

At a joint, in-person meeting with both teams, each YT study case was reviewed and a consensus reached regarding the most plausible source, given the combination of WGS and field epidemiological data. During this meeting, informal training and background information regarding the interpretation and limitations of genotyping and WGS data are presented, including discussion of the genotyping and genomic data for each case.

### Qualitative assessment

To examine the YCDC's team knowledge, attitudes and practices around genotyping and genomic services, we conducted an online, multiple-choice survey both before and after the in-person consensus meeting (see Supplementary Tables S1 and S2 for questions). At the conclusion of the consensus meeting, we also conducted a semi-structured group interview with the TB prevention and care team who completed the field epidemiology-based source identification – three nurses, a programme manager (also a nurse) and Yukon's Chief Medical Officer of Health. The interview's objective was to collect qualitative feedback on the usefulness of molecular and genomic data for the investigation of TB cases, the potential added value of MIRU-VNTR and WGS, and how this information could be used prospectively (see Supplementary Table S3 for questions). The interview questions served as prompts to structure the conversation, but all persons were free to comment, at any depth. The interview was recorded and manually reviewed. Using a thematic analysis method [21], statements were coded and categorised according to identified common themes.

### Statistical methods

We compared the insights into TB transmission provided by field-based and genomics-based teams by analysing the outcomes of each investigation at three levels of resolution – individual (i.e. did the source identified by the teams match), population-level (i.e. was the case ascribed to the appropriate transmission cluster – defined genomically) and probable location of TB acquisition (YT, BC, other province/territory or outside Canada). All statistical analyses were completed using R (v3.4.1). Agreement between results for identified source from the field- and genomic-based investigations was measured using Cohen's *κ*. The *κ* values of <0.2, 0.21–0.40, 0.41–0.60, 0.61–0.80 and 0.81–1.00 indicate poor, fair, moderate, good and very good agreement, respectively [22]. Fisher's exact test was used for comparisons of proportions. Correlations between qualitative variables, level of certainty assigned to source identification, were assessed using Spearman's *ρ*.

### Ethics

Ethics approval for this study was granted by the University of British Columbia (certificate #H12-00910).

## Results

**Fig. 1.**
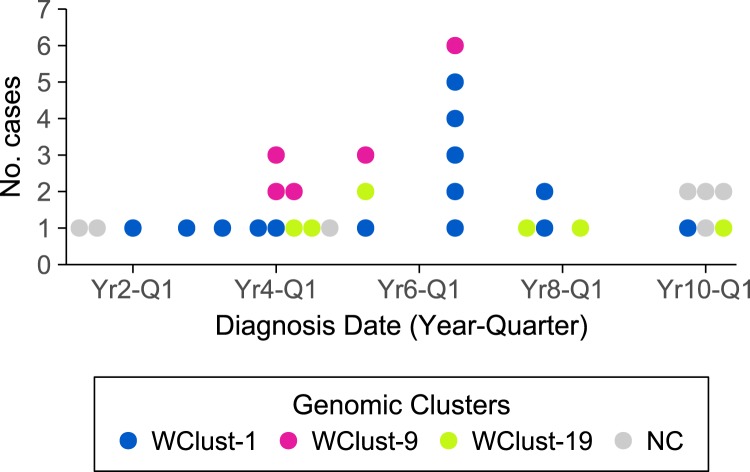
Number of tuberculosis cases by year-quarter of diagnosis over a 10-year period in Yukon, Canada. Each circle represents a single case, and colours distinguish the three large clusters identified by a combination of whole genome sequencing and traditional epidemiology. NC (Not Clustered) represents persons with *Mycobacterium tuberculosis* strains unique within Yukon.

Detailed clinical and epidemiological information, social contact data, 24-locus MIRU-VNTR genotypes and whole genome sequences were available for all 32 (100%) of the YT study cases/isolates diagnosed from 2005 through 2014. Typically, 1–2 cases were diagnosed each quarter, with the epidemiological curve (Fig. 1) showing notable peaks, each corresponding to an increase in cases matched to two of the three circulating YT-specific *Mtb* strains, as defined by WGS.

### Good agreement around clusters and location of TB exposure between methods

**Fig. 2.**
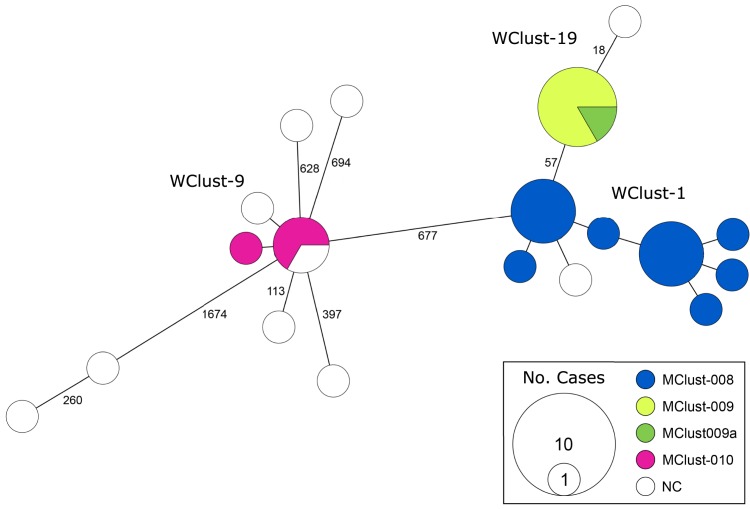
Minimum-spanning tree based on whole genome sequences of *Mycobacterium tuberculosis* (*Mtb*) isolates from the Yukon Territory (YT), Canada study population (*n* = 32). The size of each circle is proportional to the number of isolates, and circles are coloured to represent the MIRU-VNTR cluster (MClust). Isolates not matching identically at all 24 MIRU-VNTR loci were considered not clustered (NC). Whole genome sequence cluster identifiers (WClustID) are indicated for isolates clustering using a five SNV threshold. The number of SNVs between isolates with >5-SNVs is indicated along the connecting branches.

**Fig. 3.**
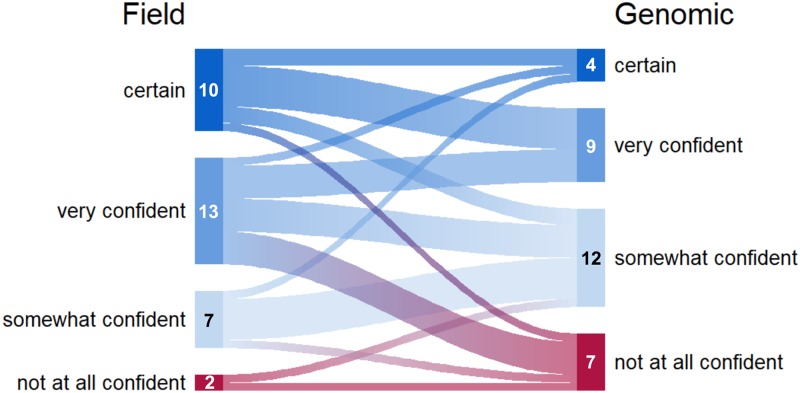
Relationship between degree of certainty assigned to each source case/location identified by field- and genomic-based methods. Link widths are proportional to the number of cases which are indicated in the margins.

Both the field-based analysis and WGS identified three large clusters (Fig. 2); however, 21 of 32 study isolates were assigned to one of these three clusters by the field-based team, while WGS placed 25 isolates into these large clusters. Three of the four discordant cases represented scenarios in which the MIRU-VNTR pattern differed from the larger clusters' patterns by a single locus – these were reported as genotypically unique MIRU-VNTR isolates in YT by the laboratory, and led the team to conclude that despite the fact the two of these three had epidemiological linkages to known YT cases, these individuals had either acquired TB from an unknown individual in BC (*n* = 1) or another province/territory (*n* = 2). The fourth discordant case had a MIRU-VNTR pattern common to both YT and BC, and while WGS placed this isolate with a genomically distinct YT sub-cluster within this group (see Fig. 3: WClust-1 in our previous work [18]), the field team classified the individual with this isolate as having acquired TB within BC based on epidemiological information.

Of the remaining seven cases in the study, both teams agreed that two were the result of reactivation of LTBI acquired abroad; both were persons born outside Canada and had *Mtb* with unique MIRU-VNTR genotypes within YT. For the five other cases, three had isolates genomically clustered with BC isolates (⩽5 SNVs), one had an isolate 26 SNVs from a BC genomic cluster and one had an isolate 18 SNVs from a cluster observed only in YT. The field team classified three of these individuals as having acquired TB in BC from an unknown source, thereby agreeing with the genomic assignment. The remaining two were hypothesised by the field team to have acquired their infection within Canada but not YT or BC – a relatively unlikely scenario according to the WGS results.

Ultimately, the two teams agreed on 26/32 (81%) locations of acquisition (Table 1), with a Cohen's *κ* of 0.68 (*P* < 0.001). Concordance was highest amongst individuals belonging to the large YT clusters and persons born outside Canada. Qualitative feedback collected at the consensus meeting indicated multiple reasons for conflicting assessments. Unique MIRU-VNTR patterns were cited as a frequent cause – both scenarios in which an isolate's MIRU-VNTR pattern was a single-locus mismatch to an existing cluster, therefore reported as unique (e.g. WClust-9, Fig. 2), and in which an isolate's MIRU-VNTR pattern was unique to YT but identical to a strain circulating in BC – as was a lack of epidemiological linkages to another YT case.

**Table 1.** Location of tuberculosis (TB) infection. For each Yukon Territory (YT) individual diagnosed with TB (*n* = 32), we show a pairwise comparison of the two methods used to identify a source. The four possible categories provided to the YT field nurses and BC Centre for Disease Control genomic epidemiologists included YT, British Columbia (BC), Other Province/Territory and Outside Canada.

Genomic epidemiology	Field epidemiology				Totals
	YT	BC	Other Prov./Territory	Outside Canada	
YT	17	1	2	0	20
BC	0	7	3	0	10
Other Prov./Territory	0	0	0	0	0
Outside Canada	0	0	0	2	2
Totals	17	8	5	2	32

### Low genomic variability within clusters limited identification of an exact source

We next examined each team's identification of a specific source for the subset of cases with a named source (*n* = 22). The two teams agreed on a likely source in 13 (59%) instances; of the nine mismatches, all but one belonged to the largest cluster, WClust-1 (Table 2). When we compared source case assignments during the in-person consensus meeting, the discussion revealed that the team using field-based data struggled with the complex social network of this cluster – many connections between individuals, while the team using WGS data were challenged by the minimal genomic diversity between YT isolates (0–4 SNVs). The presence of a minority variant in one WClust-1 case [18] divided the cluster into two genomically linked sub-clusters, facilitating source identification at the consensus meeting. While there was no strong agreement between the two team's source case assignments, the field-based methods did accurately link individuals to the correct WClust-1 genomic sub-cluster for 11 of 13 persons (85%) with only one individual linked to the incorrect genomic sub-cluster, and a second individual thought to have acquired their infection in BC due to an absence of clear epidemiological connections.

**Table 2.** Match/mismatch between methods of investigation – field- and genomic-based epidemiology – for tuberculosis source case identification, overall and by a large cluster

Characteristic	Match *n* (%)	Mismatch *n* (%)	Totals^a^
Overall	13 (59)	9 (41)	22
Large cluster			
WClust-1	5 (38)	8 (62)	13
WClust-9	3 (75)	1 (25)	4
WClust-19	5 (100)	0 (0)	5

a Excluded individuals not assigned a specific source case by field- and/or genomic-based methods (*n* = 10).

### Confidence in correct source identification varied between teams

During the in-person meeting, the genomic, clinical and CI data were combined and discussed at length to determine the most plausible source for each individual. Overall, named sources were assigned to 22 individuals, and the degree of certainty – not at all, somewhat, very confident or certain – for each inferred source identified during our independent investigations was examined. Comparing the confidence category assigned to each inferred source revealed no correlation (*P* = 0.365) between the two teams. The team using genotyping and contact data on average reported higher levels of certainty in their source ascertainment (*P* = 0.007) compared to the genomics-based team (Fig. 3). We also noted differences within and across the large clusters (Supplementary Fig. S2). The largest genomic cluster (WClust-1) had the widest distribution of confidence in source case ascertainment; conversely, participants reported higher confidence in inferred sources in the smaller clusters (WClust-9; WClust-19), particularly the team using field-based data, who reported ‘very confident’ or ‘certain’ for all source cases identified.

### Preference for genomics over genotyping

Each member of the YCDC TB programme team (*n* = 4) completed an online survey at the outset of the study in which they were asked about their role in TB prevention and care and their knowledge of genotyping methods and use in TB investigations. All respondents were engaged in direct patient care and treatment, including the collection of patient information, supervising daily medication doses, CIs and programme oversight, and three of four spend an average of >60% of their week on TB-related activities. All respondents had a background in nursing with approximately 13–35 years of experience – most of which in rural and remote communities.

Three of the four team members had heard of MIRU-VNTR prior to this study, through presentations, conferences and/or journal articles. Only one respondent reported using MIRU-VNTR information in their daily work. None indicated that they had received formal training in the use and interpretation of MIRU-VNTR in TB investigations, although three of four were aware that MIRU-VNTR data were available for their cases. No respondents reported complete confidence in using MIRU-VNTR data for their investigations, and none had used MIRU-VNTR to inform their TB investigations prior to this study.

At the conclusion of the in-person consensus meeting, a semi-structured group interview was conducted to collect qualitative feedback regarding the use of molecular/genomic data in this setting. Two main themes emerged as detailed in Box 1.

Box 1. Key themes from a semi-structured group interview with the field team regarding the use of tuberculosis molecular/genomic data

1. **The accuracy of genomics over genotyping.** The team reported that MIRU-VNTR genotyping data conflicted with the known epidemiological connections in a number of instances, whereas genomics identified clusters more closely aligned with the epidemiological data, and provided some novel insight.
  ‘*I'm liking MIRU a little less*’
  ‘*The MIRU can be helpful or not helpful*’
  ‘*To have had the WGS data, would have saved many hours of discussion – would have helped to focus the discussion by narrowing the list of potential sources*’
2. **Programme assessment.** Participants acknowledged that genomic epidemiology provided new insights into transmission patterns and saw WGS as a way to assess the effectiveness of treatment and prevention programmes, including screening and prophylaxis.
  ‘*Many of these confirmed our suspicions*’
  ‘*It was nice to know this was a reactivation and not a contact of a missed source*’
  ‘*Small case load means few people working on TB, and we need to focus limited resources on *the highest risk contacts.'**
  ‘*… prophylaxis could have prevented the cluster*’

Overall, participants viewed genomic epidemiology as a useful tool to streamline investigations, particularly in differentiating LTBI reactivation from the recent transmission, but not essential to their current practices, instead noting it would be most useful for programme assessment. The team found that WGS results were useful for confirming probable source cases and ruling out local transmission. MIRU-VNTR data were cited as a source of frustration where it did not align with the epidemiology. Improved communication around how to interpret closely related MIRU-VNTR patterns, as well as the limitations of genotyping, was strongly recommended.

In a post-meeting follow-up online survey (Supplementary Table S2), respondents reiterated the themes from the in-person group interview by highlighting their preference for WGS over MIRU-VNTR, with qualitative feedback such as ‘*WGS provides a clearer picture than MIRU-VNTR of what is happening in terms of transmission*’. Additionally, respondents noted that WGS highlighted some gaps in knowledge or what may have been missed during contact tracing, supporting the idea of using WGS towards programme assessment. When asked if they felt more confident using WGS data following this study, all stated that they were considerably more confident and would like to have genomic data for all cases. The team also indicated they would be open to further training in the interpretation of genomic data, with in-person training preferred over an instruction manual or instructional videos.

## Discussion

In this study, we investigated the added value of using genomics in a setting with rich field epidemiological data, as well as the knowledge, attitudes and practices around the use of molecular and genomic data for TB case investigations. Comparing the traditional approach of inferring TB transmission from genotyping and CI data to the use of genomics with limited case-level data revealed that WGS appeared to improve identification of connections between cases at a high level, such as cluster membership, but that the data from CIs was integral to identifying source cases at an individual-level, particularly within large clusters.

In certain settings, genotyping by 24-locus MIRU-VNTR has been reported as having high discriminatory power and good concordance with known epidemiological linkages [23–25]. However, technical issues with particular loci, rendering them untypable, can make cluster assignment challenging – these patterns are often assigned their own unique identifier, and obscure the potential linkage between isolates [26–28]. *Mtb* isolates with single-locus mismatches have been shown to be linked by both epidemiology and genomics [6, 23, 29–31]. In our study, these falsely ‘unique’ MIRU-VNTR patterns complicated the interpretation of CI data, with the true nature of clustering only revealed through the higher-resolution genomic approach. Given that WGS may not be available to all TB programmes, we recommend that laboratories reporting MIRU-VNTR data include information not just on identical patterns, but also closely related patterns that might suggest a larger cluster. Our survey results also indicated that there is a substantial gap in training end-users to interpret genotyping data, suggesting that laboratories might consider including some interpretive commentary on their genotyping reports beyond simply a pattern and a cluster identifier. Where WGS data are available and are provided to TB programmes, it will be essential to consider the format in which data are provided [32], and to offer training and support by genomic epidemiologists.

As expected, genomic data coupled with basic clinical and epidemiological data were able to identify clusters and infer some potential sources, but it was only when they were combined with extensive CI data that a more comprehensive picture of TB transmission began to emerge. This highlights the importance of engaging the laboratory, public health nursing and epidemiology staff in the joint interpretation of genomic epidemiology data. In remote Northern settings with extensive person-to-person transmission [1, 33], the minimal genomic variation observed means that data from CIs are integral to understanding local epidemiology, and that enhanced investigation questionnaires, as recently used in a UK study, can establish epidemiological connections between individuals that would have otherwise not been linked [25]. During discussions with the YCDC public health team, they noted that, had WGS data been available during CIs, more focused questioning likely would have uncovered some missed connections and would have helped to confirm/refute tenuous linkages, saving time and resources. Discussions also revealed a strong preference for WGS over MIRU-VNTR to support CIs, and identified programme assessment as an important secondary use for WGS data. For such a remote setting, YT has a relatively short turnaround times for most TB test results due to efficient specimen transport networks; however, the ability to inform CIs using genomic data remains limited in all settings by the necessity of WGS using DNA extracted from culture. Although there has been progress in obtaining WGS results directly from specimens [34], these methods are not yet robust enough for routine use.

Our study also identifies the importance of sharing molecular epidemiology data across jurisdictional boundaries. Genotyping results are routinely reported at the provincial/territory level, but information on the presence of a pattern in another jurisdiction may not always be provided. Here, the YCDC team did not have access to molecular data from BC cases prior to this study, and were unaware that six cases with a MIRU-VNTR pattern unique to YT were actually members of genotypic clusters with BC cases [18]. This is an issue commonly faced in the tracking and tracing of TB even in urban settings, where populations frequently move between jurisdictions. Tools that allow public health personnel to identify matched cases across health service care boundaries, such as Ontario's OUT-TB Web [35], can be used to increase communication between TB programmes.

A major strength of the present study was the availability of a small, well-characterised population, particularly with the long service of several of the nurses involved in the study who have considerable experience within the community. A limitation of the comparison between investigation methods was that the team using data from CIs could make connections between culture-positive and -negative cases; however, the molecular and genomic analyses were limited to culture-positive cases and may have resulted in missed linkages between individuals. A further limitation could be social desirability bias in the qualitative feedback. To address this, we asked some of the same questions through the anonymous online post-meeting survey and found the same sentiments in regard to usefulness and training around genotype/genomic data. However, we recognise there may still be bias in this format and this should be taken into consideration when interpreting qualitative results. For example, the relatively small number of local public health personnel responsible for CI in this setting may limit the generalisability of the survey findings.

## Conclusions

Our study highlights the need to better integrate laboratory, clinical and epidemiological data to more comprehensively describe TB transmission in a given setting, including using higher-resolution genomic approaches where possible, providing a better interpretation of MIRU-VNTR data when WGS is not available, and bringing individuals together for collaborative discussion of cases and clusters. Through a genomics-informed, enhanced CI approach, we believe that TB programmes might better focus their resources and avoid missed opportunities for intervention, thereby limiting new transmissions. For this to occur, communication is key. Given the dynamic and complex nature of genomic and CI data, regular review of cases through in-person meetings and training in interpretation is recommended. Genomics also has the potential to aid in TB programme evaluation, and as the technique becomes more commonplace, TB laboratories and prevention and care programmes must work together to jointly assess the impact of this emerging epidemiological approach.

## Supplementary material

For supplementary material accompanying this paper visit https://doi.org/10.1017/S0950268820000072. click here to view supplementary material
