# Supplementary material for: Comparison of routine field epidemiology and whole genome sequencing to identify tuberculosis transmission in a remote setting
Source: Epidemiol Infect. 2020 Feb 4;148:e15. doi: 10.1017/S0950268820000072 (PMC7019559; doi:10.1017/S0950268820000072)
Supplement: Supplementary file 1 [file S0950268820000072sup001.pdf]

Epidemiology and Infection

Comparison of Routine Field Epidemiology and Whole Genome Sequencing to Identify Tuberculosis Transmission in a Remote Setting

J.L. Guthrie, L. Strudwick, B. Roberts, M. Allen, J. McFadzen, D. Roth, D. Jorgensen, M. Rodrigues, P. Tang, B. Hanley, J. Johnston, V.J. Cook, J.L. Gardy

Supplementary Material

Supplementary Figure S1. MIRU-VNTR cluster summary example.

**MClust-008**
This cluster belongs to the **Euro-American** lineage, and the 24-locus MIRU-VNTR pattern has been seen **40** times in British Columbia from **2005** through **2014** in the following BC health service delivery area(s): **South Vancouver Island, Vancouver, Fraser East, Fraser North, Fraser South, Northwest**<br><br>The overall demographics of this MIRU-VNTR cluster (BC & YT): 62% male, median age (years) = 45 (IQR: 35-55) and is 95% Canadian-born.

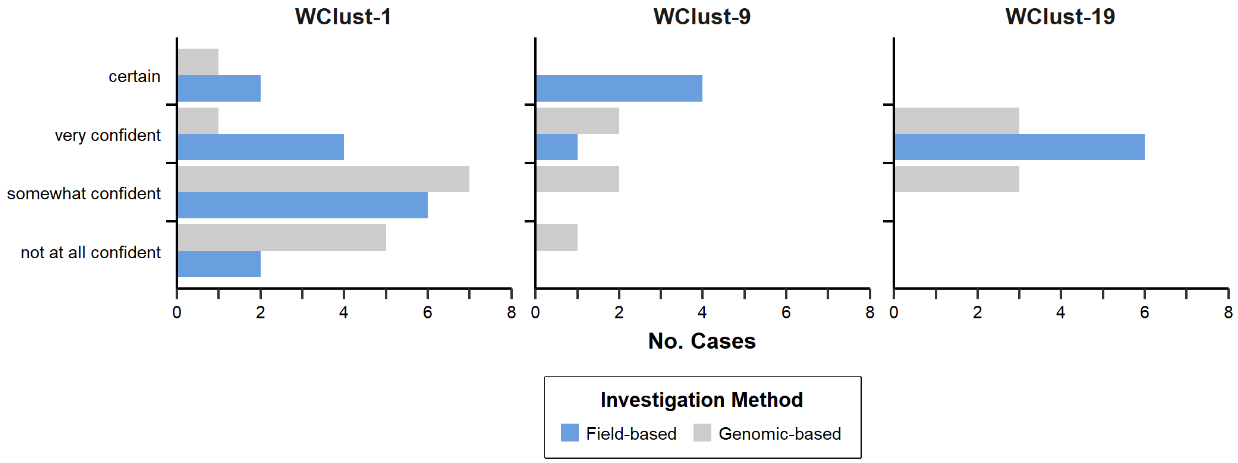

Supplementary Figure S2. Frequency of certainty categories assigned for each source identified, divided by cluster.

Supplementary Table S1. Online survey questions administered pre-meeting.

Questions

A. Basic Information

1. Which tasks related to tuberculosis are generally part of your job? (*Select all that apply*)
   - Supervising daily patient medication doses
   - Collecting contact information directly from patients
   - Writing notes on each patient encounter
   - Entering information into Panorama
   - Case management
   - Outbreak investigation
   - General program oversight
   - None of the above
2. What are your working hours?
   - Full time
   - Part time
   - Other (please specify)
3. Approximately how long have you worked in the area of TB?
   - < 1 year
   - 1-5 years
   - > 5 years
4. Do your daily activities include anything other than TB?
   - Yes
   - No
5. What proportion of your average week do you spend on TB-related work?
   - 0%-19%
   - 20%-39%
   - 40%-59%
   - 60%-79%
   - 80%-100%

B. TB Genotyping Knowledge

6. Prior to this study had you heard of TB genotyping (*i.e. MIRU-VNTR/spoligotyping/RFLP*)?
   - Yes
   - No
7. Which genotyping methods have you heard of? (*Select all that apply*)
   - RFLP/fingerprinting
   - MIRU-VNTR
   - spoligotyping
8. Through what means have you been exposed to information about genotyping? (*Select all that apply)*
   - daily work
   - presentations
   - conferences
   - journal articles
   - co-workers
   - reports
   - other
9. Have you ever received formal training (e.g. course, workshop, user guide) in the use of genotyping data for investigations? (*e.g. confirm/refute transmission, guide contact tracing*)
   - Yes
   - No
10. Prior to this study were you aware that genotyping data (MIRU-VNTR) was available for your TB patients?
   - Yes
   - No

C. Current Process

11. Prior to this study, have you used MIRU-VNTR genotyping data in your investigations? (*e.g. confirm/refute transmission, guide contact tracing*)
   - Yes
   - No
12. Prior to this study, what have you used MIRU-VNTR genotyping data for? (*Select all that apply*)
   - Confirm clusters and links between cases
   - Refute clusters and links between cases
   - Identify unknown links between cases
   - Justify extending contact investigation
   - Investigate potential false positive TB diagnosis
   - Don't know
   - Other (please specify)
13. Prior to this study, how often do you use MIRU-VNTR genotyping data in your case management or outbreak investigation?
   - Never
   - For few cases
   - For about half of cases
   - For many cases
   - For every case
14. How confident are you in using MIRU-VNTR genotyping data in your investigations? (*e.g. confirm/refute transmission, guide contact tracing*)
   - Novice – not at all confident
   - Average – somewhat confident
   - Expert – completely confident

Supplementary Table S2. Online survey questions administered post-meeting.

Questions
**A. Future Processes**
1. How often would you like to use MIRU-VNTR genotyping data in your TB case management or outbreak investigations?<br>• Never<br>• For few cases<br>• For about half of cases<br>• For many cases<br>• For every case
2. After completion of this study do you feel more confident using MIRU-VNTR genotyping data in your investigations?<br>• Not at all<br>• Somewhat<br>• Considerably
3. How often would you like to use whole genome sequencing (WGS) data in your case management or outbreak investigation?<br>• Never<br>• For few cases<br>• For about half of cases<br>• For many cases<br>• For every case
4. After completion of this study do you feel more confident using whole genome sequencing (WGS) data in your investigations?<br>• Not at all<br>• Somewhat<br>• Considerably
5. Would you like to receive training in interpretation of TB genotyping and genome sequencing?<br>• Yes<br>• Maybe<br>• No
6. What format would you prefer for training? (*Select all that apply*)<br>• In-person workshops<br>• User guides<br>• Online videos<br>• Other
7. Reason you do not want training?<br>• Not part of my job<br>• Not valuable<br>• No time<br>• Other
8. Do you have any comments about the survey or the study that you would like to make? We value all input.

Supplementary Table S3. Interview questions used to guide discussion.

Category	Questions
Impact	- Overall, do you think that genotyping and/or genome sequencing data improved your understanding of transmission dynamics in Yukon? - Do you think that you would have changed your approach to any investigations if you had genotyping and/or genome sequencing data at the time of investigation? - What proportion of cases do you feel that genotyping data and/or genome sequencing data would be helpful? - Overall, do you think that there is added value in genotyping and/or genome sequencing Yukon's TB isolates?
Future Processes	- How should the BCCDC Public Health Laboratory, clinicians, and contact investigation teams communicate genotyping and/or genome sequencing information to YCDC? *Data:* raw data or analyzed/interpreted data *Format:* case-level reports, regular summary reports, regular teleconferences, phone calls as needed - After reviewing the genotype cluster descriptions for Yukon's MIRU-VNTR clusters we'd like to get your feedback. - Did you find the cluster descriptions useful? *(Useful / Not very useful / Useless)* - What other information would you like to see in the cluster descriptions? - Other feedback?
